# Comparative evaluation of pediatric patient comfort, time, and preference between digital scans and rubber base impressions: crossover study randomized controlled trial

**DOI:** 10.1186/s12903-025-07192-8

**Published:** 2025-12-14

**Authors:** Asmaa Mohamed Gamal, Yasmine Ahmed Mortada Abd Elfatah

**Affiliations:** Pediatric Dentistry and Dental Public Health Department, Faculty of Dentistry, Assiut University, Assiut, Egypt

**Keywords:** Digital impression, Intraoral scanner, Patient comfort, Polyvinyl siloxane (PVS)

## Abstract

**Objective:**

This study aims to assess pediatric patient comfort, time, and preference between digital scans and rubber base impressions.

**Materials and methods:**

Randomized crossover study in which thirty patients aged 7–11 years underwent an intraoral scan and rubber base (PVS) impressions of both arches, separated by one week. The sequence of impression procedures was determined using block randomization with allocation concealment through sealed envelopes. The two techniques’ impression times were compared. Patients were asked which one they preferred. The Visual Analogue Scale (VAS) was administered to the patient to determine comfort and gag reflex.

**Results:**

80% of patients preferred a digital scan to a rubber base, which was significant (*P* = 0.000). The digital scan took a shorter time than the rubber base, with a difference (4.16 ± 0.12; 95% CI: 3.92 to 4.39, *p* = 0.000). Comfort was significantly higher for digital impressions, with a difference (2.8 ± 0.56; 95% CI: 1.66 to 3.94, *p* = 0.000), while gag was smaller for digital impressions (0.7 ± 0.29; 95% CI: 0.09 to 1, *p* = 0.009).

**Conclusion:**

Digital impressions were better tolerated by children, offering greater comfort, reduced gag reflex, and shorter procedure time compared to conventional impressions. These findings suggest that digital impressions may be a more favorable option for children in clinical practice.

**Trial registration:**

The ClinicalTrials.gov identifier NCT06833385 13-02-2025.

**Supplementary Information:**

The online version contains supplementary material available at 10.1186/s12903-025-07192-8.

## Introduction

An impression is defined as a negative reverse copy of the surface of an object. It is a necessary step in space maintainer appliances, orthodontic diagnosis, and treatment planning. Conventionally, dental trays were used to take the impression, and plaster models were obtained. “However, there are certain problems with this traditional approach, such as the need to preserve the models, the difficulty of pouring and trimming plaster casts—which requires significant laboratory work—and the need for a person to transfer the impression from the clinic to the lab. Additionally, one of the main problems with the conventional use of trays is the gag reflex experienced by the patient [1, 2].

The recent use of digital impressions, which provide a three-dimensional image of the teeth and surrounding structures, allows dentists to assess the degree of dental crowding and development within the dental arches. This examination becomes easier and more accurate with a digital scan, as it employs software and virtual dental models. The software can measure arch length and discrepancies, as well as track progress in space regaining with minimal effort [3–5].

Dentists are increasingly using intraoral scanners as study models. Digital scanners can provide precise impressions while also reducing gag reflexes and patient discomfort, and offering time savings and ease of use [6].

The child’s comfort during impression taking and the duration necessary are crucial considerations, as it is widely recognized that the longer and more difficult the impression, the greater the anxiety and stress experienced by the child [7]. Several studies have been conducted on the accuracy of digital scanner models; however, investigations comparing impression techniques with regard to comfort, preference, and time have only been carried out in young adults or adult patients [8].

Hence, the objective of this study was to assess pediatric patient comfort, time, and preference between digital scans and rubber base impressions. The null hypothesis stated that there was no statistically significant difference among the digital and rubber base impression (PVS) techniques with regard to patient preference, gag reflex, comfort, and procedure time.

## Materials and methods

### Ethical considerations

The Ethics Committee of the Faculty of Dentistry Assiut University approved the study and obtained informed consent prior to the start of the analysis (reference number 17–2025−0002). The clinical procedures were registered under the ClinicalTrials.gov identifier NCT06833385. The research adhered to the ethical guidelines set forth in the World Medical Association’s Declaration of Helsinki for studies involving human participants [9]. Participants and their parents/guardians were provided with detailed information about the procedures—including potential discomforts, risks, and benefits—and informed consent was obtained from their legal guardians before the study began.

### Study design

The current study employed a randomized crossover design, in which each participant underwent both digital scanning and rubber impression procedures. The two impression techniques for both dental arches were evaluated in two separate sessions, with a one-week interval between procedures for each participant.

Throughout all phases of the study, the design and reporting adhered to the Consolidated Standards of Reporting Trials (CONSORT) to ensure clinical trial transparency [10].

### Sample size

As demonstrated in the study conducted by Bosoni et al. (2023) [11], 75% of patients preferred digital impressions. Assuming a null hypothesis of a proportion of 50% of oral lesions and an alternative hypothesis of 75%, and employing the G*Power Statistical Power Analysis program (version 3.1.9.4) for sample size determination [12]. A total sample size (*n* = 30) will be sufficient to identify a medium effect size (g = 0.25), with an actual power (1 - β error) of 0.8 (80%) and a significance level (α error) of 0.05 (5%) for a two-sided hypothesis test.

### Participants

Thirty patients, aged 7 to 11 years, who visited the Pediatric Dentistry Department at the Faculty of Dentistry, Assiut University, for space maintainers or orthodontic treatment were included in the study. Recruitment took place during the period from February to May 2025.

Inclusion criteria for patient selection:

- There was no prior experience with either digital scanning or conventional rubber impressions.

Exclusion criteria for patient selection:

1. Children with uncooperative behavior.
2. Patients having syndromes or systemic diseases.
3. Patients with cleft lip and palate.
4. Dental abscesses, gingival bleeding.
5. Restricted mouth opening.

### Randomization, allocation concealment and blinding

The sequence of the two impression procedures was determined using block randomization, ensuring that 15 patients underwent conventional rubber impressions first, while the remaining 15 patients began with intraoral scanning. Randomization was conducted using the block randomization technique with sealed envelopes.

An independent researcher, not involved in the study, generated the allocation sequence employing computer-generated randomization (https://www.sealedenvelope.com/simple-randomiser/v1/lists**)** and ensured allocation concealment through sealed envelopes. Participants were assigned to their respective groups by opening serially numbered, concealed envelopes immediately before the impression procedure.

Blinding of the operator and patients was not feasible due to the nature of the impression procedures.

### Clinical procedure

Digital scan was performed with (3Shape Trios4). To perform this scan, four steps were followed sequentially: patient registration, mandibular, maxillary, and bite scans. According to the routine of this digital scan, missed areas appeared in green color. Missed areas were scanned without the impression being repeated, especially using this method suggested by the manufacturer company. For every patient, the time taken to complete the digital impression was measured from the initiation of patient registration until the completion of mandibular, maxillary, and bite scans. The duration was recorded in minutes using a stopwatch. Any verbal explanation or isolation placement was not included in the recorded time. The total time required to complete the digital impression for one patient was in average 3.50 min.

The poly vinyl silicate (PVS) impressions were obtained using (Aquasil Soft Putty, Dentsply Sirona, Germany), mixed as per the manufacturer’s instructions after tray selection (stainless steel stock tray) with suitable size. The first impression was performed using putty consistency which requiring approximately 2 min in patient mouth, followed by a wash impression with light body which took about 1.5 min in patient mouth. The impression was then left to set as per the manufacturer’s instructions. For every patient the total time taken to complete the rubber impression were also four: tray selection, impression of the mandibular arch, impression of the maxillary arch, and bite registration with dental wax in one piece.

The two impression procedures were conducted in two sessions for both arches, with a 1-week interval between the two procedures. Both impressions for the dental arches were completed by a single qualified operator (AGMZ). Another operator (YAMAF) prepared the PVS impression material and recorded the impression times for both procedures.

### Outcomes

**Fig. 1:**
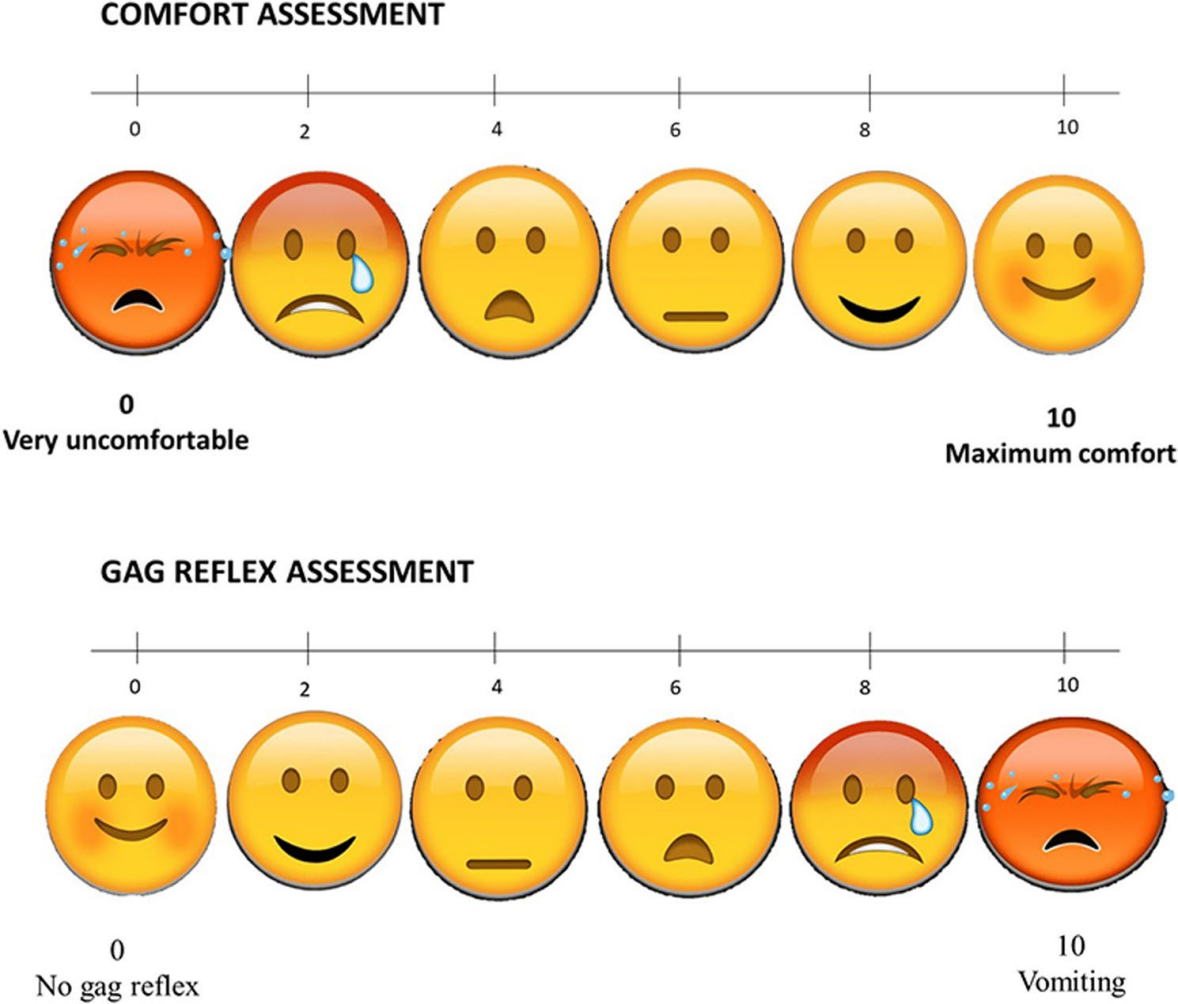
Visual Analogue Scale with Wong–Baker scale to evaluate patient comfort and gag reflex in the two impression methods

The main goal of the study was to assess which of the two procedures patients preferred. The patients were asked which of the two impression techniques they preferred. Secondary outcomes were the period of the procedure, comfort, and gag reflex. Patients were given a questionnaire that included a visual analogue scale (VAS) for comfort and the gag reflex. The VAS scale ranged from 0 to 10. The Wong-Baker Scale was also used for interpretation (Fig. 1). In terms of comfort, 0 corresponds to extremely uncomfortable and 10 to maximum comfort. For gag response, 0 corresponds to no gag reflex and 10 to vomiting. Finally, the total chairside time for each impression technique was recorded using a digital stopwatch, starting from the initiation of the impression procedure until its completion.

### Statistical methods

Statistical analysis will be done using available software (SPSS, Chicago, IL, USA). Numerical data will be described using counts and percentages. The data will be compared using the chi-squared test. *P* ≤ 0.05 will be employed to establish statistical significance.

## Results

**Fig. 2:**
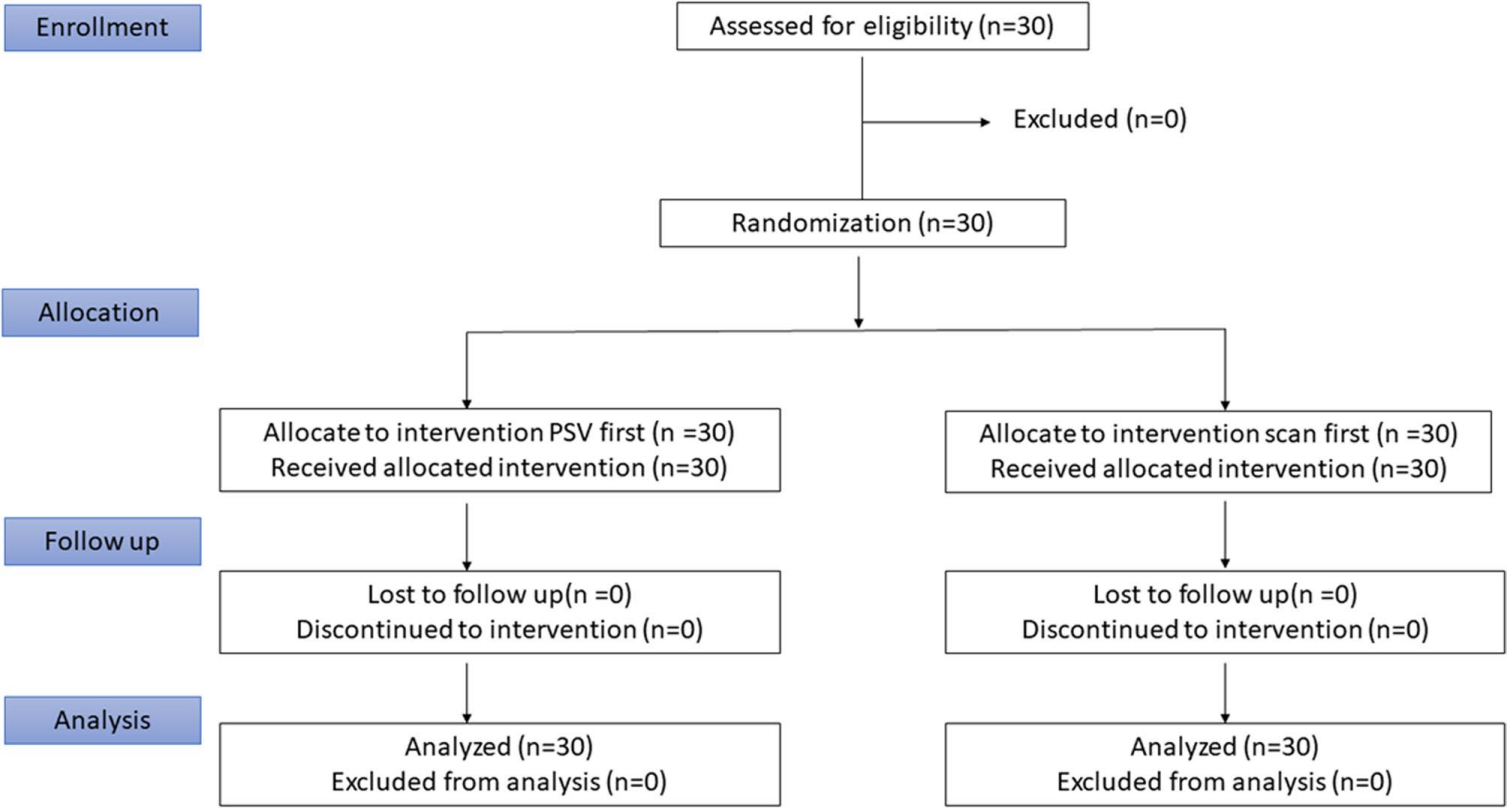
Consort flow diagram

Thirty participants were recruited from February to May 2025. No dropouts occurred, and the planned protocol was followed exactly (Fig. 2).

Thirty participants were randomly allocated to the two impression techniques. Among the participants, 11 (36.7%) were aged 7–9 years, while 19 (63.3%) were in the 9–11 years age group, with no difference in patients’ distribution (*p* = 0.704) (Table 1).

**Table 1:** Gender and age distribution of participants (chi square test)

Gender Age	Male	Female	Total	X^2^ value	*P* value
Age 7–9	5	6	11 (36.7%)	0.144	0.704 ns
Age 9–11	10	9	19 (63.3%)		
Total	15 (50%)	15 (50%)			

Significance level *p*≤0.05, *ns *non-significant

According to patients’ preference, a total of 24 patients (80%) expressed a preference for digital impressions, whereas 6 patients (20%) favored the PVS impression technique. This difference in preference was statistically significant (*p* ≤ 0.05) (Table 2).

**Table 2:** Comparison of patients’ preference (chi square test)

Patient preference	Digital	PVS	X^2^ value	*P* value
Cases	24 (80%)	6 (20%)	21.6	0.000*

Significance level *p*≤0.05, *significant

Comparison between digital scans and PVS impressions in terms of patient comfort, gag reflex, and procedure duration is presented in Table 3.

**Table 3:** Descriptive statistics and comparison between digital scans and PVS impressions groups regarding comfort and gag reflex (Mann–Whitney U test), and time (t-test)

	Groups	Mean	Std. Dev	Median	Difference				t test	*P* value
					Mean	Std. Dev	95% C.I. lower	95% C.I. upper		
Comfort	Digital	1.40	2.06	0.00	2.80	0.56	1.66	3.94	Z=−3.93	0.000*
	PVS	4.20	1.44	4.00						
Gag reflex	Digital	0.00	0.00	0.00	0.70	0.29	0.09	1.31	Z=−2.61	0.009*
	PVS	0.70	1.30	0.00						
Time	Digital	3.56	0.38	3.42	4.16	0.12	3.92	4.39	t= −35.53	0.000*
	PVS	7.71	0.36	7.55						

Significance level *p*≤0.05, *significant

Comfort variable was recorded as (1.4 ± 2.06, median 0) in the digital scan group. On the other hand, comfort variable was (4.2 ± 1.44, median 4) in the PVS group, with a difference of (2.8 ± 0.56; 95% CI: 1.66 to 3.94) between the two groups. The difference between groups was statistically significant (*p* = 0.000).

Gag reflex variable was recorded as (0 ± 0, median 0) in the digital group, in comparison to (0.7 ± 1.3, median 0) in the PVS group, with a difference between groups of (0.7 ± 0.29; 95% CI: 0.09 to 1.31). The difference among groups was statistically significant (*p* = 0.009).

Impression time was significantly shorter for the digital group (3.56 ± 0.38) than in the PVS group (7.71 ± 0.36), with a difference between groups (4.16 ± 0.12; 95% CI: 3.92 to 4.39). The difference between groups was statistically significant (*p* = 0.000).

## Discussion

In recent decades, full-arch intraoral recording using intraoral scanners has gained popularity due to its reported sufficient accuracy [13, 14] and great applicability in orthodontic and padiatric specialties [15]. This investigation sought to assess padiatric patient comfort, time, and preference between digital scans and rubber base impressions.

In this research, conventional impressions were done employing PVS, as it is considered one of the most accurate and dimensionally stable impression materials reported in the literature, with a shorter setting time, instead of alginate impression materials, which were used in a previous study as conventional [16]. While alginate has been found to have the lowest surface detail accuracy and dimensional stability when relative to elastomeric impression materials, it is still widely used due to its ease of use and cost-effectiveness [17].

The present study used a combination of both the Visual Analogue Scale (VAS) and Wong-Baker Face Scale (WBCS) for the assessment of patient comfort and gag reflex, as they are considered preferred methods for obtaining child self-reports and were shown to be appropriate tools for assessment among children aged 3 to 14 years [18].

​Patients were selected during the mixed dentition stage, typically between the ages of 7 and 11 years — a period when orthodontic assessments and treatment are commonly initiated. At this age, children are generally mature enough to express their preferences regarding dental procedures, including the choice of impression technique.

Our study, which assessed different techniques of impressions collected from the same patient at 7-day intervals (a crossover study), has made patient comparisons simpler. In some studies, all impressions were taken in a single visit [6, 19], which may lead to a carry-over effect [20, 21] and impact the study’s results.

The null hypothesis that there was no statistically significant difference among digital and conventional impression techniques regarding patient preference, comfort, gag reflex, and procedure time was rejected.

Based on available information, this is the first clinical trial that compared digital and rubber base impressions. Therefore, there is no similar literature to compare with the present results. Thus, the total outcome was compared to that obtained in previous studies that used alginate as the conventional impression.

In the current study, 80% of participants preferred digital impression and 20% preferred rubber base impression. This result aligns with the majority of prior studies in which patients preferred the digital impression approach [20, 22–24]; in others, the preference was for the conventional alginate impression process [6], and in others, there were no differences [19].

This finding might be attributed to the digital impression’s decreased invasiveness compared to the rubber base impression, as shown by the better outcomes in comfort and gag response. Along with less invasiveness, there was a considerable decrease in impression period.

The current study’s findings are consistent with those of Mangano et al. [25], Yilmaz and Aydin [5], and Burhardt et al. [20], who documented a higher preference for intraoral scanning (100%, 75%, and 51%), and differ from the findings of Grunheid et al. [6], who found that 73% of patients preferred the conventional impression method and 27% preferred the digital method.

In the present study, 20% not preferring digital scanning may be owing to the size of the scanner head.

In the current study, the total chairside time needed for digital impressions was (3.56 ± 0.38), which was significantly less than the time needed for rubber base impressions (7.71 ± 0.36). The shorter chairside time of digital impressions may have made it preferred with pediatric patients.

This finding goes in accordance with several previous studies. Bosoni et al. [11] also documented shorter chairside time for digital impression. Burhardt et al. [20] and Mangano et al. [25] documented a significantly reduced time for conventional impression with alginate, which contrasts with these results. Conversely, there was no discernible difference in the overall impression time among the two techniques, according to Yilmaz and Aydin [5].

This variation in results could be attributed to the reduction of scanning times due to the enhancing of the quality of operator experiences (hand and digital skills) in digital scanning. Patients recruited in the current study were between the ages of 6 and 11, at a stage of dentition before the eruption of the second molars. The scanning process could have been sped up by the lack of the permanent second molars, since they were in a location that was hard for the scanner tip to reach. The shorter duration of digital scanning decreases chairside time, improving cooperation in young patients.

In this study, there was a statistically significant distinction among the two impression methods in terms of comfort and gag reflex. This similar to the outcome of Mangano et al. [25], Glisic et al. [26], Yilmaz and Aydin [5], and Bosoni et al. [11].

The current study’s limitations were the lack of intra-rater agreement on the VAS, and the intraoral scanner employed in this investigation is not the most recent version available from the manufacturer. Another limitation was that just one operator with experience in both impression procedures was employed. Given that operator experience may have an impact on patient comfort, the scope of this study might be expanded by incorporating operators with different levels of experience. In addition, the relatively small sample size may affect the strength of the conclusions. Other important factors include potential patient-related variables that may influence perception and preference.

## Conclusions

Digital scanning provides greater patient comfort, with less gag response and reduced procedure time compared to rubber base impressions. This finding highlights the potential of digital technology in dentistry. Therefore, this study contributes to optimizing patient care through the adoption of modern, patient-friendly technologies.

## Supplementary Information

Supplementary Material 1.

## Data Availability

The datasets generated and/or analysed during the current study available from the corresponding author on reasonable request.

## Abbreviations

PVS: Polyvinyl siloxane
VAS: Visual analogue scale
CONSORT: Consolidated Standards of Reporting Trials
WBCS: Wong-Baker Face Scale
